# Informing the development of antipsychotic-induced weight gain management guidance: patient experiences and preferences – qualitative descriptive study

**DOI:** 10.1192/bjo.2024.725

**Published:** 2024-08-01

**Authors:** Ita Fitzgerald, Erin K. Crowley, Ciara Ní Dhubhlaing, Sarah O'Dwyer, Laura J. Sahm

**Affiliations:** Pharmacy Department, St Patrick's Mental Health Services, Dublin, Ireland; and Pharmaceutical Care Research Group, School of Pharmacy, University College Cork, Cork, Ireland; Pharmaceutical Care Research Group, School of Pharmacy, University College Cork, Cork, Ireland; Pharmacy Department, St Patrick's Mental Health Services, Dublin, Ireland; and College of Mental Health Pharmacy, Burgess Hill, UK; Department of Medicine, St Patrick's Mental Health Services, Dublin, Ireland; Pharmaceutical Care Research Group, School of Pharmacy, University College Cork, Cork, Ireland; and Pharmacy Department, Mercy University Hospital, Cork, Ireland

**Keywords:** Antipsychotics and antipsychotic-induced weight gain, schizophrenia, health services research, guideline, patient preferences

## Abstract

**Background:**

Antipsychotic-induced weight gain (AIWG) is a substantial contributor to high obesity rates in psychiatry. Limited management guidance exists to inform clinical practice, and individuals with experience of managing AIWG have had no or minimal input into its development. A lack of empirical research outlining patient values and preferences for management also exists. Recommendations addressing weight management in psychiatry may be distinctly susceptible to ideology and sociocultural values regarding intervention appropriateness and expectations of self-management, reinforcing the need for co-produced management guidance. This study is the first to ask: how do individuals conceptualise preferred AIWG management and how can this be realised in practice?

**Aims:**

1. Explore the management experiences of individuals with unwanted AIWG. 2. Elicit their values and preferences regarding preferred management.

**Method:**

Qualitative descriptive methodology informed study design. A total of 17 participants took part in semi-structured interviews. Data analysis was undertaken using reflexive thematic analysis.

**Results:**

Participants reported that clinicians largely overestimated AIWG manageability using dietary and lifestyle changes. They also reported difficulties accessing alternative management interventions, including a change in antipsychotic and/or pharmacological adjuncts. Participants reported current management guidance is oversimplified, lacks the specificity and scope required, and endorses a ‘one-size-fits-all’ management approach to an extensively heterogenous side-effect. Participants expressed a preference for collaborative AIWG management and guidance that prioritises early intervention using the range of evidence-based management interventions, tailored according to AIWG risk, participant ability and participant preference.

**Conclusion:**

Integration of this research into guideline development will help ensure recommendations are relevant and applicable, and that individual preferences are represented.

## Background

Antipsychotic-induced weight gain (AIWG) is commonly cited as one of the most distressing treatment side-effects and a frequent cause of non-adherence.^1,2^ Clinically significant weight gain has been linked to almost all antipsychotics^3^ and contributes significantly to the two- to three-fold higher obesity rates seen among those with a severe mental illness (SMI).^4^ Consequently AIWG and its impact on the risk of cardiometabolic disease contributes appreciably to the poorer physical health,^4^ reduced number of healthy years lived^5^ and the 10- to 20-year premature mortality seen among those with an SMI.^6,7^ Given the personal, social and healthcare costs, effectively managing AIWG is a key priority for patients, clinicians and policymakers.

However, successfully managing AIWG is challenging.^8^ Although inter-individual variability in the prognosis of AIWG is an important factor, limited management guidance for clinicians invariably increases management complexity.^7,9,10^ Although a very limited number of recommendations exist within larger guidelines,^11–13^ a rigorously developed clinical practice guideline dedicated to AIWG management is notably absent.^10^ This includes guidance informed by consultation with varying stakeholder groups, and recommendation development to align with international standards.^14,15^ The absence of a quality guideline is an important barrier to timely, systematic and equitable patient access to evidence-based AIWG management across cohorts and settings.^16,17^

Calls are increasing for standardised guidance dedicated to AIWG management.^10,18^ Aside from concerns regarding inequitable individual access to management interventions, reasons underpinning such calls include the need for guidance for clinicians regarding the role of non-pharmacological and pharmacological management interventions specific to AIWG management.^12,16,19^ This is particularly important in the case of dietary and lifestyle interventions, where evidence of efficacy diverges from that demonstrated in the general population.^16^ Accumulating evidence addressing novel anti-obesity medications, including glucagon-like peptide 1 (GLP-1) analogues, in psychiatric populations also requires due consideration.^20^ Guideline development, however, requires evidence addressing criteria beyond intervention effectiveness. This includes individual values and preferences for management interventions and views regarding the acceptability, feasibility and transferability of ensuing recommendations.^15,19,20^ One challenge in developing relevant and applicable AIWG management guidance is the lack of empirical research assessing these criteria. To date, the scope and endorsement of management recommendations have largely been informed by expert opinion of guideline development groups with no or minimal patient input.^12,21,22^ The prognosis of AIWG is extensively variable,^3^ and thus patient preferences and priorities for management are likely similarly variable.

Healthcare providers can (often unconsciously) endorse stereotypical assumptions and stigmatising attitudes about those living with obesity.^23,24^ Obesity management among those with an SMI may be particularly liable to such implicit biases, where the experience of a ‘dual stigma’ has been described by those living with both obesity and an SMI.^25,26^ Recommendations addressing weight management in those with an SMI may be distinctly susceptible to ideology and sociocultural values regarding the appropriateness of interventions, including use of anti-obesity medications and expectations of self-management.^27^ These concerns reinforce the need for explicit patient engagement during guideline development.

This study is the first to ask: How do patients conceptualise preferred management of AIWG and how can this be realised in practice? Qualitative research methods are particularly helpful in ascertaining individual values and preferences for treatment interventions, while also acquiring evidence addressing other key guideline decision-making criteria.^15^ The World Health Organization recently reinforced the importance of considering qualitative evidence during guideline development to ensure that the views, needs and preferences of people with lived experience are considered during recommendation development and in designing implementation processes.^15^

### Aims and objectives

**Fig. 1:**
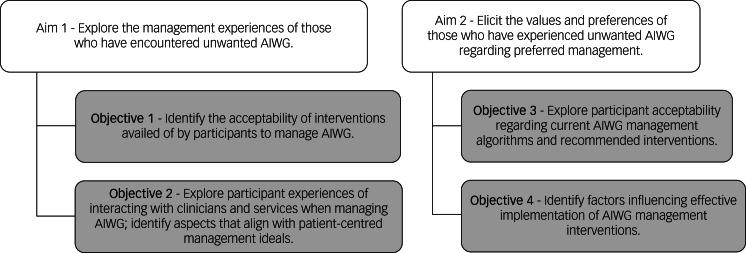
Overview of study aims and objectives. AIWG, antipsychotic-induced weight gain.

Figure 1 outlines the study aims and objectives. Eliciting acceptability of previous management experiences will inform feasibility and implementation considerations, including the impact of wider social, contextual and personal factors on effective AIWG management.

## Method

The consolidated criteria for reporting qualitative health research checklist (COREQ) informed research reporting and are contained within the Supplementary Appendix available at https://doi.org/10.1192/bjo.2024.725.^28^

### Study design and setting

Qualitative descriptive methodology was employed, given that study aims were not to provide an increasing theoretical, conceptual or highly abstract understanding of the topic, but rather to provide a descriptive summary of the informational content of the data that could be easily understood and implemented by stakeholders.^29,30^ Quality standards consistent with the application of qualitative descriptive methodology were adhered to. Philosophical assumptions underlying the methodology have been previously outlined.^29^ Participants attending out-patient services of a psychiatric secondary care setting in the Republic of Ireland were included. Although recruited through one centre, participants were explicitly asked to reflect on all experiences of managing AIWG to capture varying experiences over time and across settings. Participants were primarily recruited via advertisement where participants were asked to respond to advertisements placed in out-patient settings. Clinicians could also highlight the study to potentially relevant individuals. Five participants were known to one researcher (I.F.) through a prior clinical relationship.

The study involved semi-structured interviews lasting 45–60 min. Interviews took place primarily online because of COVID-19 restrictions in place during project planning. Following the subsequent lifting of restrictions, two interviews took place in person. Interviews were audio recorded only and supplemented with field notes taken during interviews. All interviews were conducted by one researcher (I.F.), a female senior pharmacist working within the recruiting centre, with expertise in conducting qualitative interviews. All procedures involving participants were approved by St Patrick's Mental Health Services (SPMHS) Research Ethics Committee (Protocol 02/22). All procedures complied with ethical standards of the relevant national and institutional committees on human experimentation and with the Helsinki Declaration of 1975, as revised in 2008. Written informed consent was obtained from each participant prior to enrolment.

### Participant recruitment

Table 1 outlines inclusion and exclusion criteria.

**Table 1:** Study inclusion and exclusion criteria

Inclusion criteria	Exclusion criteria
Adults ≥18 years of age. Those with a diagnosis of a severe mental illness (SMI) including bipolar affective disorder, schizophrenia (or other enduring psychotic illness) or major depression and who were prescribed an antipsychotic for any period of time. Those with experience of unwanted antipsychotic-induced weight gain.	Those receiving in-patient care. Cases where weight gain was not primarily because of antipsychotic treatment. Those prescribed an antipsychotic for an illness other than an SMI.

Purposive sampling guided the sampling strategy whereby participants with diverse experiences of SMI and AIWG and its management were purposely recruited. We also purposely included participants with varying sociodemographic characteristics to increase credibility and transferability of results. This included age, gender, employment status and living supports. Furthermore, concepts arising from data analysis following early interviews drove subsequent recruitment to test emerging concepts. Thus, participant sample size was not determined a priori. Recruitment cessation was based on the concept of ‘data sufficiency’ i.e. sufficient data were available to meet study aims.^31,32^ Data sufficiency was conceptualised as consensus among researchers that a sufficiently detailed and rich aggregate account of AIWG management experiences and preferences for management was reached.

### Data collection

An interview guide was drafted with reference to existing literature and study aims. The guide was reviewed by individuals within a local Service User Advisory Network (SUAN), representing those with lived experience of both SMI and AIWG management. The guide was amended based on their feedback before study commencement, and thus was not piloted. The interview guide was also updated iteratively throughout data collection to incorporate questions addressing emerging concepts. The final version is contained in the Supplementary Appendix. Verbatim transcription of interview audiotapes and redaction of identifying information was undertaken by one researcher (I.F.). Transcript verification was offered to all participants. Participant sociodemographic data were collected including information on age, gender, diagnosis and duration of diagnosis, living arrangements, employment status, duration of diagnosis and body mass index (BMI).

### Data analysis

Thematic analysis was conducted according to the principles of Braun and Clarke, specifically their reflexive approach,^33,34^ involving six phases: familiarisation with data, generating initial codes, generating themes, reviewing potential themes, defining and naming themes, and producing the report. Initially three researchers (I.F., E.C. and L.S.) double-coded anonymised transcripts independently using NVivo (Release 1.7.1 for Mac). Both semantic and latent codes were developed, largely inductively.^33^ Subsequently, a codebook was developed and used by all members of the research team to discuss emerging findings, develop themes and discuss additional recruitment needs. Themes were developed separately for each aim for greater clarity in result presentation. To enhance study rigour, negative cases analysis was conducted whereby cases that appeared to contradict or challenge emerging themes were actively pursued, and theme development evolved accordingly. Once agreed upon by all researchers, participants were offered the opportunity to member-check themes for accuracy and validity. Final themes were reviewed and updated accordingly.

## Results

Seventeen participants were interviewed. Participant clinical and sociodemographic information is contained in Table 2.

**Table 2:** Summary characteristics of participants (*n* = 17)

Characteristic	Summary
Female	76%
Age in years Mean (range)	51.5 (26–66)
Body mass index (BMI) Mean (range)	31.2 (22.5–40.6)
Employment status	
Employed	65%
Unemployed/state support	35%
Living arrangements	
With family (independent of family of origin)	76%
With family of origin	18%
Alone	6%
Primary diagnosis	
Schizophrenia or associated subtype	24%
Bipolar affective disorder	35%
Major depression	41%
Duration of mental illness	
<1 year	6%
1–5 years	29%
5–10 years	6%
>10 years	59%

### Aim 1: Explore the management experiences of those who have encountered unwanted AIWG

**Fig. 2:**
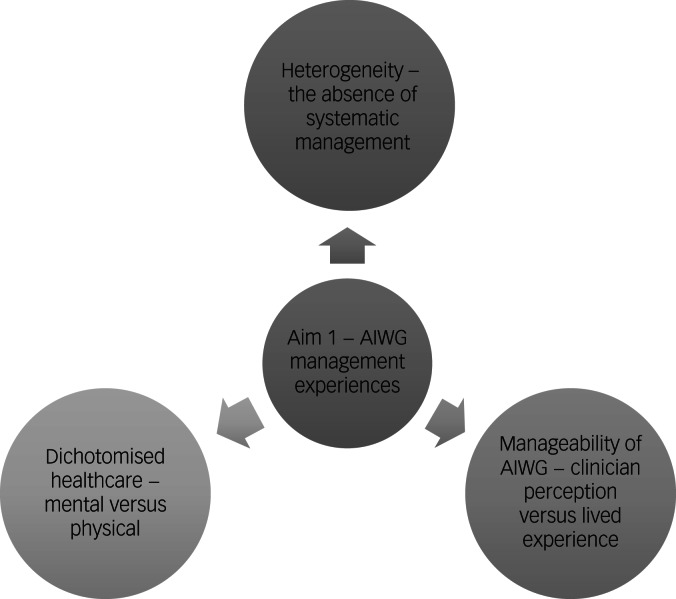
Thematic map of the identified themes relating to antipsychotic-induced weight gain (AIWG) management experiences.

An overview of themes outlining participants’ experiences of managing AIWG can be found in Fig. 2.

### Heterogeneity – the absence of systematic management

Significant heterogeneity in experiences of AIWG management was apparent at the level of the participant (e.g. responsiveness to intervention), clinician (e.g. experience of a collaborative management) and psychiatric service (e.g. participant access to exercise interventions). A systematic approach to management appeared largely absent, with participant reports of experiences ranging from minimal support to shared management between psychiatric and endocrinology services. Weight gain secondary to antipsychotic treatment not being systematically seen as the responsibility of psychiatric services to collaboratively manage was identified as a significant source of heterogeneity. Participants across varying settings reported a prevailing narrative that the burden of responsibility of AIWG management was left with them to navigate unsupported.

P16 – ‘*There was just a complete emphasis on personal responsibility and very little appreciation from staff of what you were dealing with, because maybe they had never had that experience of having those intense cravings. With several different doctors, never once was anything other than diet and lifestyle recommended*.’

Absence of collaborative management responsibility was also perceived by participants through the absence of preparative information on antipsychotic weight gain liability and management methods, continued clinician recommendations to implement self-led behavioural interventions, independent of success of prior trials, and clinician reluctance to intensify management.

P6 – ‘*A lot of the answers that were being offered, well go away and try this yourself and you know, if you try this yourself, come back to us, sure some people don't gain the weight at all, or that's a pity you just happen to be in the group that do*.’

P7 – ‘*I haven't been given any advice, whether it's alternative medication, or training, or anything like that … I'm left to row my own boat*.’

Whereas a largely self-led management approach was acceptable among participants with comparatively minor weight increases, this approach led most to feel that failure to effectively manage AIWG was within their control.

P13 – ‘*I always felt like a bit of a failure really and having to apologise and justify my weight gain*.’

Beyond minor weight increases, participants reported that behavioural changes alone were insufficient to manage AIWG. Where pharmacological adjuncts were available, participants with experiences of moderate or severe AIWG reported finding these interventions as more helpful than lifestyle modifications alone.

P11 – ‘*I would not have been able to lose that weight on my own … it would have taken years to lose what I've lost with Ozempic*®’.

Participants reported that use of metformin specifically was helpful in plateauing continuing AIWG and in reducing food cravings, both of which were valuable outcomes to participants in addition to weight reversal.

P5 – ‘*I think the metformin obviously helps because I don't have that urge to come in and eat lots of stuff*.’

P11 – ‘*It [metformin] didn't really have a massive effect, but it keeps things stable … it does play a role in weight management in terms of antipsychotics. It's better for prevention more than an actual therapy to reverse*.’

However, participants with experiences of severe AIWG reported that changing to a lower-risk antipsychotic treatment or stopping antipsychotic treatment had the most significant impact upon AIWG manageability, including effectiveness of behavioural changes.

P14 – ‘*They switched the olanzapine to Seroquel® and although it's not perfect, it's much, much better, much less weight gain … for somebody who would be on olanzapine, that [lifestyle] course would be inappropriate because you wouldn't be able to get out of it what you needed*’.

Many participants described being unaware of the option of changing antipsychotic or the use of pharmacological adjuncts to manage AIWG.

P3 – ‘*You saying those three things, the diet and lifestyle, switching to another medication or trying an add-on, I was not aware that that existed. I didn't even know that there were alternatives to quetiapine*.’

Participants with experiences of different psychiatric services reported that access to intensified management was dependent upon care being provided by clinicians or teams who adopted a holistic approach.

P6 – ‘*Now I'm under the care of [service] and they reviewed my medication, and the team did listen to me about the weight gain being a factor and part of my unwellness and they swapped the olanzapine for quetiapine to try and change that*.’

Among those with experiences of collaborative management, engagement with multi- and interdisciplinary clinicians experienced in AIWG management was identified as particularly valuable.

P5 – ‘*It was great when I was able to speak with a pharmacist and I felt, I think I have a voice now, somebody can hear what I'm concerned about*.’

P13 – ‘*There's no judgement, I just feel able to relax around [endocrinologist] and just be honest and open about how I feel … I kind of hang on to that, really valuable*.’

**Fig. 3:**
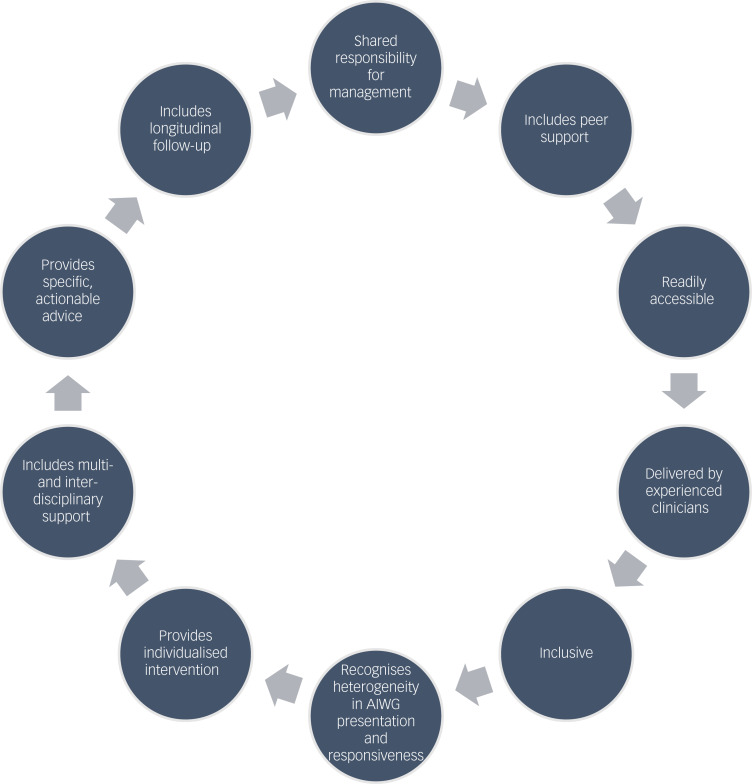
Participant descriptions of aspects of effective antipsychotic-induced weight gain (AIWG) management interventions.

In cases of positive experiences of managing AIWG, aspects of management approaches reported as effective and preferable are summarised in Fig. 3.

### Manageability of AIWG – clinician perception versus lived experience

All participants spoke about the uniqueness of managing weight gain induced by antipsychotic treatment specifically, including intensity of food cravings and loss of appetite control. Table 3 gives an overview of participants’ experiences of managing antipsychotic-induced appetite increases in cases of both mild AIWG (P5, P9) and severe (P1, P13) AIWG.

**Table 3:** Descriptions of experiences of weight gain and appetite increases secondary to antipsychotic treatment

Participant 1	‘It's a hunger you just can't do anything about, you know, food doesn't, food doesn't satisfy it. So, it's insatiable, you just can't. I couldn't find way really, any other way really other than be hungry. I could eat twice the amount of dinner and you're still as hungry as when you started.’
Participant 5	‘Often at night when I was going to bed, I was sneaking in food to the bedroom, which I never did in my life before and it was like, I kept saying “What is going on here?”’
Participant 9	‘I mean you are out of control in your thoughts when you're psychotic but then you're given this drug that taints these thoughts, but then your physical self is out of control because you're just filling that need within you [to eat].’
Participant 13	‘Zyprexa® seems to just make me balloon out … I just couldn't get enough of the food, I just couldn't get enough. I was just completely out of control, there was no satiety. There was no, I just couldn't get enough. It was fatty foods; it was carbs and as much as possible.’

Participants described a disconnect between what they perceived to be the clinicians’ perception of their ability to manage AIWG through self-intervention, and their experience of AIWG management without additional clinician intervention.

P6 – ‘*You're trying to get across, I'm really trying my best here you know, but feeling that maybe they didn't think you were making enough of an effort*.’

P16 – ‘*There was the sense that this was simple if you only follow this certain number of steps. The problem is that those steps, in the context in which people find themselves, can be incredibly difficult, if not impossible, to follow*.’

Many participants described difficulties in implementing behavioural changes due to contextual factors, including antipsychotic side-effects, fluctuating psychological health and physical consequences of AIWG. Participants reported that the impact of these variables was frequently unaccounted for in management advice received.

P2 – ‘ *People say shake it off and try and go out and get a bit of exercise, but when I was up at the weight I was, it was 95 kg I went up to, that's 49 kg up to 95 kg. I found it hard to go out walking for exercise because physically my knees couldn't take it because of my body weight*.’

Among participants with experiences of being on low-, medium- or high-risk antipsychotics relating to risk of AIWG, an absence of differentiated clinician advice was reported. This contrasted starkly with participant experience of manageability when prescribed different antipsychotics.

P16 – ‘*When you're not on these drugs or when you're on less of them, or when you're on ones that don't have quite that effect, you're just simply not as hungry. Your appetite isn't as intense. I don't know if I would be able to do all those things [lifestyle changes] as well or stay at a healthy weight if I was on a larger dose, if I was on the medicines that I was on when I gained a lot of weight*.’

The difference between clinician perception and participant experience of AIWG manageability potentially contributed to difficulties participants encountered in accessing management interventions requiring clinician collaboration, including changing antipsychotic and accessing pharmacological adjuncts.

P4 – ‘*Even my own doctor when I said to him about this this injection [semaglutide] … he knew everything I'd been through, why is he making me wait when he knows this is going to be an issue?*’

P16 – ‘*Is there still something of a stigma as in why that's [pharmacological adjuncts] the absolute last resort, is it because of the potential side-effects or is it because we feel that people should be handling this themselves?*’

Continual clinician recommendations to implement self-led behavioural changes could worsen participants’ sense of isolation and internalised stigma, and reduce the likelihood of future help-seeking due to anticipated stigmatising interactions.

P10 – ‘ *It leaves me feeling kind of isolated as well because how it looks is that I am not making an effort to improve my position and then I don't want to see people and be judged for it … being overweight and having mental health issues, I just don't want to be perceived as lazy*’.

Where participants perceived that clinicians displayed an understanding of the unique challenges of managing weight gain induced by antipsychotic treatment, participants reported reduced psychological distress.

P6 – ‘*To hear from a professional that actually this is really hard, this can happen, it has happened in your case, there are things you can do to lessen it, but you may always have a bit of a battle on your hands … just to take some of the shame away*’.

### Mental versus physical healthcare – a false dichotomy

Dichotomisation of physical and mental healthcare within psychiatric services was an additional barrier that participants described in accessing AIWG management support. Many participants reported that clinicians minimised concerns about AIWG liability or occurrence.

P3 – ‘*Any time I brought it up … it would be kind of invalidated in that like, don't be so silly, look at how well you're doing, don't be worrying about gaining a few pounds*’.

Some participants reported perceiving there to be, or explicitly being presented with, a choice between physical and mental health when discussing the impact of AIWG with clinicians, particularly when requesting a change in antipsychotic.

P11 – ‘*My experience over the 12 years was there's your medications, take them and be happy. The whole “Would you rather be skinny and unstable or overweight and stable?” and you're just like, come on*!’

Participants described a bidirectional relationship between their physical and mental health and the impact that unmanageable AIWG could have on their recovery from mental illness.

P4 – ‘*OK, my mental health is bad, but this [weight gain] is a mental health problem too … it's a huge thing for me*’.

Many participants described self-reducing or stopping antipsychotic treatment to reverse AIWG.

P10 - ‘*I came off that [olanzapine], I was having a hard time managing my mental health, but I'd lost a lot of the weight, and it was kind of down to … yeah, I, I stopped it*’.

### Aim 2: Elicit the values and preferences of those who have experienced unwanted AIWG regarding preferred management

**Fig. 4:**
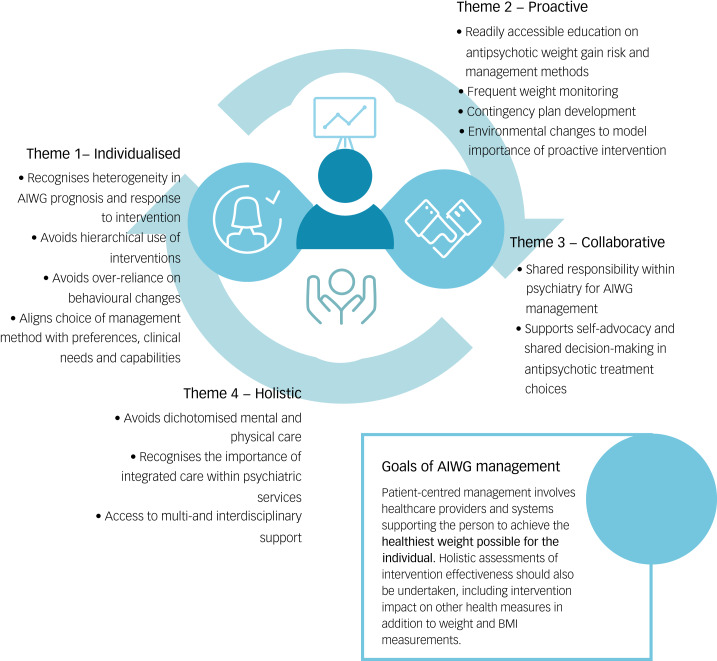
Participant view of pillars encompassing improved antipsychotic-induced weight gain (AIWG) management practices.

Figure 4 provides an overview of the four themes of individual-centred AIWG management described by participants.

### Individualised management

Participants were presented with the current stepwise AIWG management approach, beginning with behavioural/dietary and exercise interventions as the standard management intervention, followed by consideration of changing antipsychotic and subsequent use of pharmacological adjuncts e.g. metformin, if the preceding options are ineffective.^11,13^ Participants reported that recommendation of behavioural interventions as a uniform first-line intervention oversimplified the complexity of managing AIWG.

P14 – ‘*It's easy enough to say you need to lose weight, try diet and lifestyle, but you're saying that at a time when a person is at a low … it has to be very structured and what sick person is going to be able to do that?*’

Participants collectively spoke of individual-centred management requiring a more individualised approach.

P9 – ‘*You could talk about all the different options and then together with the patient decide what's the best for them, rather than the robotic steps, so to make it a more individual approach*.’

Individualised management included avoidance of uniform treatment pathways endorsing hierarchical intervention use. One repeated argument against this management approach was heterogeneity in AIWG prognosis and response to intervention.

P16 – ‘*If somebody comes and they've gained three or four stone over the course of a year, that would seem to be a much different issue than if somebody had gained a few pounds. The same answer shouldn't be the case*.’

Those with a personal history of obesity were particularly concerned about recommendations supporting hierarchical intervention use, and the assumption that all management interventions are equally applicable to presenting individuals.

P1 – ‘*I wouldn't really think that option one [diet and lifestyle] alone will work in practice. It might work for a week but it's not sustainable, particularly if you are big to begin with, I can certainly put on a stone in a week*.’

Disagreement with uniform recommendation of behavioural changes as first-line management was also present among participants who were severely unwell at antipsychotic initiation.

P6 – ‘*If you were dealing with psychosis, if somebody has been very ill, they may not be able to make diet and lifestyle changes. In their case, the tailoring may involve more preventative use of metformin because they just may not be in that position to make those decisions for themselves that really require being very proactive at a time when you are recovering*.’

Other participants expressed concern that recommendations endorsing diet and lifestyle interventions lacked specificity and reinforced their perception that management was primarily their responsibility.

P13 – ‘*You can feel totally dismissed if someone says “Oh diet and exercise,” you just end up going, OK, here we go again. Someone that doesn't give you direct, specific, measurable and achievable advice … you just feel like you are getting fobbed off again*’.

P16 – ‘*Option one [diet and lifestyle] is a fob-off, definitely … you feel like a fly and the person is trying to swat you away. What does this mean, diet and lifestyle, what should a person's first step be?*’

Participants who preferred initial use of behavioural interventions recommended that these be structured interventions and resourced within psychiatric services, and that their initial use should be agreed collaboratively, following due consideration of alternative management options.

P3 – ‘*It's getting some support on the diet and the lifestyle, but it is more understanding you have options, for me it's that, it's understanding that you have options*.’

P16 – ‘*There could be a structured programme in place, things that people can follow … a shared burden in terms of diet and lifestyle*’.

Participants said that a preferred management option would be for a change of antipsychotic to be explicitly discussed with them, given the significant differences they experienced in manageability when prescribed different antipsychotics.

P10 – ‘*I would probably say switch to lower-risk first and then diet and lifestyle*.’

Participants viewed pharmacological adjuncts as relevant and acceptable interventions, and requested that these be offered earlier than currently endorsed.

Interviewer – ‘*Do you think we should be offering other options like metformin earlier in treatment or not?*’

P4 – ‘*100%. That would be like if you had cancer, the chemotherapy or radiation or whatever is making you sick, there is immediately an anti-sickness drug … it's not something you wait to be told about*’.

P15 – ‘*Well, that would be the option I would want because then I'd know I'm sticking with the medication that's working and trying to help with the cravings*.’

Many participants, particularly those with experiences of AIWG secondary to high-risk antipsychotics and those with a personal obesity history, expressed a preference for preventative use of pharmacological adjuncts.

P5 – ‘*If it's a known drug for weight gain, it [metformin] should be offered at the beginning … rather than wait six months down the road and then somebody feeling awful about the drug and deciding they want to come off it*’.

### Proactive management of AIWG

Participants cited proactively addressing AIWG as crucial to effective management and highlighted that recommendations addressing prevention and early intervention were missing from current guidance. Contingency planning was one suggestion to operationalise proactive management.

P6 – ‘*Where are the contingency plans so when it happens, as is often the case, it's not an if, it's a when it happens … that word proactive is what's really the key*.’

Use of markers or thresholds to prompt early consideration of intensified management by clinicians was also suggested.

P6 – ‘*If somebody's BMI has radically increased maybe there should be some marker that identifies that person as somebody that the health service gets involved with quicker*.’

Participants wanted to be supported in being proactive, and highlighted that information and education on AIWG risk are important in their preparedness for management.

P15 – ‘*I didn't realise that the medication I was on could cause weight gain …. if I had been educated a little bit more about what my medication side-effects were, I would have been able to handle it a bit better. I probably would have considered my diet and lifestyle a bit better*.’

Information on AIWG risk and management interventions being accessible and readily available was also highlighted consistently.

P8 – ‘*Definitely more education on the management options, I think the earlier in the process the better*.’

Accessible information was also described as important in reducing potential power asymmetry in discussions between patients and clinicians, which could be worsened by experiences of overweight and obesity.

P4 – ‘*Knowledge is powerful … if you have the background, it makes you feel like you're being listened to, and you're being recognised*’.

Participants also spoke of the importance of services and environments modelling the importance of proactive weight management.

P17 – ‘*It highlights for you the importance of your own management of the situation and the fact that there is something to be cognisant of, something to be aware of*.’

### Collaborative management

Participants reported an expectation that psychiatric clinicians should be involved in AIWG management and reported that for management to be effective, it needs to be seen as collaborative. This includes being initiated within services where antipsychotics are prescribed.

P6 – ‘*I think they have a shared responsibility … I think it can't be entirely on them [psychiatrists], but they have expertise and with that comes an authority and a responsibility that the patient doesn't have … if it was seen as such, it would be less easy to dismiss concerns*’.

A fundamental aspect of collaborative AIWG management endorsed by participants was shared decision-making in the choice of antipsychotic treatment, particularly the choice to continue the causative antipsychotic. This should include explicit engagement with individuals’ assessment of the risks of continued AIWG versus the risks of changing antipsychotic.

P15 – ‘*When I started to notice the weight gain, I was well enough to try another medication*.’

P17 – ‘*I would have changed from Seroquel® completely if I had known there was an alternative*.’

### Holistic service provision

Participants described the importance of a holistic approach to care within psychiatric settings. This included integrated care models addressing physical and mental health needs, given the burden of risk factors participants faced for living with overweight or obesity.

P6 – ‘*That dichotomy of, this is the psychiatric services and then you go over here for the physical services, if it's more integrated from the get-go I just think you wouldn't feel abandoned and there would be a much greater chance of you staying compliant with the medication which is for the psychiatric side of things and your well-being in general would be better*.’

Integrated care would also allow for more prompt access to clinicians experienced in weight management in acute care settings to align with participant preferences for AIWG management.

P8 – ‘*Earlier in the process to actually engage with the dietician and get advice to guide you in the hospital, because I was kind of working that out myself*.’

Where participants had experiences of individualised behavioural interventions being provided by experienced clinicians, their acceptability increased.

P13 – ‘ *… referred me to a dietician along the line and that was terrific, as that was really specific and helpful advice from someone who knows, that's their whole area of expertise*’.

Participants also requested longitudinal weight management supports within psychiatric services.

P1 – ‘*I think the big difference would be to formally have some kind of continuation of the supports that work. So, maybe a check in after three months and a check in after six months*.’

Among those with experiences of severe AIWG, integration of endocrinology services into psychiatry settings was advocated to improve access to intensified supports, including anti-obesity medications and psychological support.

P6 – ‘*Going to the endocrinologist and being listened to and being started on the drug [semaglutide] and even having the empathy of the endocrinologist who kind of, not kind of, but did understand how difficult it is to lose this weight, that was really reassuring. They understood that obesity is a lot more difficult to beat, more than sometimes the GP or the psychiatrist understood*.’

Participants also expressed a preference for assessment of management effectiveness to look beyond weight measurement alone and towards holistic assessments of health.

P16 – ‘*Should our aim really be to maximise a person's well-being, their mental and physical well-being and to what extent can that be done in a way that allows people to be different shapes and sizes while being still healthy*?’

**Fig. 5:**
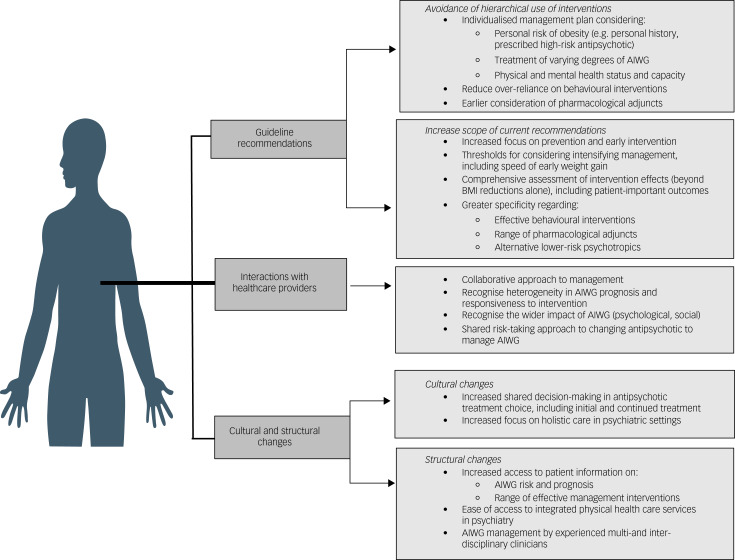
Overview of guideline and strategic changes required to support development and implementation of individual-centred AIWG management. AIWG, antipsychotic-induced weight gain.

An overview of changes suggested by participants in management guidance, alongside cultural and structural changes required within services to support implementation of patient-centred AIWG management, is contained in Fig. 5.

## Discussion

This study is the first to explore several key decision-making criteria relevant to the development of high-quality AIWG management guidance among a purposely diverse lived experience group. Feedback was sought regarding the relevance and scope of current AIWG management guidance^11,12^ alongside contextual factors that may help or hinder recommendation implementation. Acceptability and feasibility of management interventions and practices across a range of contexts was elicited. As in general weight management,^35^ heterogeneity in the responsiveness of AIWG to any intervention was reported by participants. Despite the availability of varying management interventions,^16^ participants were unaware of, or encountered, difficulties accessing interventions. Participants reported that clinicians largely overestimated the manageability of AIWG through behavioural changes and underestimated the physical and psychological impacts of AIWG.^36^ In the cases of positive management experiences, these were largely attributed to proactive clinicians who afforded participants access to collaborative and holistic care. Thus, an absence of a systematic and equitable approach to AIWG management was observed here. Collectively, these experiences reinforce the need for more effective AIWG management guidance and implementation supports.

### Current AIWG management guidance – what works and what is missing?

Participants reported that current guidance is oversimplified, lacks the specificity and scope required, and endorses a homogenous management approach to an extensively heterogenous side-effect, both in presentation and manageability. Significant changes in AIWG management guidance was called for by participants because of the lack of applicability and transferability across individuals and contexts. Suggested changes include expansion of the recommendation scope to address prevention and early intervention, and assessment of intervention effectiveness to align with patient-important outcomes. Participants emphasised that AIWG is a unique cause of weight gain, often in unique contextual circumstances, and requires an equally distinctive approach to management. Improved recommendations in AIWG management recognise diversity among individuals in the initial risk and subsequent trajectory of AIWG and allow for intervention use to be tailored towards the risk of overweight or obesity, individual physical and mental health capabilities, and their treatment preferences. Thus, participants strongly disagreed with uniform and hierarchical treatment algorithms. Participants expressed a preference for all management options to be considered collaboratively, ideally early in the experience of AIWG, and as part of contingency planning when prescribing medium- or high-risk antipsychotics. Those at high risk of AIWG generally expressed a preference for more intensive early intervention.

#### Behavioural/dietary and lifestyle changes

Participants largely disagreed with endorsement of uniform recommendation of behavioural changes as first-line AIWG management. Barriers to implementation included the impact of mental illness and antipsychotic treatment on motivation, cognition and capacity, intensity of food cravings and the speed of AIWG in at-risk individuals. Lack of resources within psychiatric services to support implementation and experiences of differential efficacy when used alongside high- versus low-risk antipsychotics were also barriers to uniform endorsement. In line with results of interventional research demonstrating wide-ranging and often clinically insignificant effect sizes associated with many dietary and lifestyle interventions,^16,36,37^ participants reported, with exception, that many of their attempts to manage AIWG with dietary and lifestyle interventions were ineffective. Thus, participants expressed a preference for guideline recommendations regarding dietary and lifestyle changes to be reflective of the evidence base in AIWG management. This includes outlining interventions associated with the largest effect sizes, those deemed ineffective and their comparative efficacy relative to other recommended interventions.^16,36,37^ Avoidance of erroneous recommendation of population-level weight management advice and continued recommendations to implement non-specific, or previously ineffective, behavioural changes were highlighted as important, given the potential impact on internalised weight stigma. Strategies to support people in effectively implementing behavioural interventions were provided and included longitudinal provision within psychiatric services and involvement of peer-support networks.

#### Pharmacological management – switching antipsychotic and pharmacological adjuncts (metformin, topiramate, GLP-1 receptor agonists)

In contrast to its relegated role in current guidance,^11^ participants endorsed the use of pharmacological adjuncts as acceptable interventions. Among those at high risk of obesity, due to treatment or personal history, their preference was for pharmacological adjuncts to be offered as preventative interventions. Participants placed a high value on their use to maintain weight and low resource cost associated with oral adjuncts. Earlier consideration of pharmacological adjuncts was also valuable to participants as it avoided risks associated with changing antipsychotic. Evidence for preventative and treatment roles of pharmacological adjuncts is now increasing and aligning with patient preferences for earlier use.^17^ However, participants also valued being informed about the differential risk of weight gain across antipsychotics and the option of switching to manage AIWG, particularly among participants prescribed high-risk antipsychotics. Although much of the intervention research and subsequent guidance in AIWG management is based on efficacy of interventions to reverse AIWG,^11,12,38^ participants suggested novel use of pharmacological adjuncts and assessment of additional outcomes not previously considered, including reduction in food cravings and weight stabilisation.

### Functional AIWG management guidance

Participants cited patient-centred AIWG management as being proactive, individualised, holistic and collaborative. Suggestions to support implementation of these tenets included changes in clinician management guidance, but also changes in clinician education and the model of service delivery in psychiatric settings. Participants discussed the need for greater support implementing interventions that target individual behaviour change, but also highlighted the multilevel structural determinants that may lead to, and/or perpetuate, the absence of patient-centred AIWG management in psychiatric settings. This included stigmatising attitudes among clinicians regarding participants’ motivation to manage AIWG, the absence of accessible patient information to inform collaborative decisions about antipsychotic treatment choices and availability of anti-obesity medications, and the absence of integrated physical healthcare services to support holistic care and early intervention. Participants requested greater clinician education on the uniqueness of weight gain induced by antipsychotic treatment, specifically interference with homeostatic mechanisms mediating appetite control and the heterogenous nature of its severity.^39^

Given its impact on AIWG prognosis,^40^ participants described a fundamental aspect of managing AIWG being collaborative decision-making in antipsychotic treatment choices. The decision to change antipsychotic involves consideration of risk by both prescribers and patients. Participants described the weighting of risks differently from their treating clinician. Given their professional training and the role of legislative and regulatory frameworks in informing service delivery, psychiatrists may prioritise minimising safety and relapse risks following a change in antipsychotic. However, most participants here associated continuing a weight-gaining antipsychotic with greater risk, and led to many stopping treatment themselves in an attempt to reduce AIWG. Participants expressed a preference for a shared risk-taking approach to decisions to change or to continue the choice of antipsychotic treatment, and described the need for cultural and structural changes in psychiatry to support their active engagement in treatment decisions. Participants valued accessing multi- and interdisciplinary clinicians when managing AIWG, particularly in managing obesity. Access to obesity management clinicians has been identified as a facilitator of greater ownership of obesity management in psychiatry.^4,41^ Co-produced guidance between psychiatric and endocrinology professions is one novel way to initially address this need, given the current absence of integrated care models in psychiatry.^42^ Co-produced guidance would also facilitate the integration into psychiatry of new paradigms and practices within weight management in general medicine. This includes person-centred management guidance,^42^ and addressing the neurobiological underpinnings of obesity,^43^ the latter of which has also been associated with AIWG risk.^44^

We have produced a framework for guideline developers to consider when constructing patient-centred AIWG management guidance. Aside from use of psychotropic medications, those living with an SMI face a disproportionate number of risk factors for experiencing obesity. This includes high rates of sedentary behaviour, poverty and stress, and weight bias among clinicians.^22^ To holistically support weight management in psychiatric settings, applying a wider lens of analysis of contributing factors is required. Implementing effective weight management strategies should include consideration of structural interventions that address the multitude of factors within psychiatric cohorts and settings that perpetuate high obesity rates and suboptimal management practices.

### Limitations

Despite intentional recruitment efforts, there is less male than female representation in this study. Results may be less reflective of male AIWG management experiences and preferences, although we are somewhat reassured by the fact that gender has not been demonstrated to meaningfully impact AIWG risk and trajectory.^40^ We did not have the opportunity to include a culturally diverse participant group which may limit result transferability. Five participants were professionally known to the researcher conducting interviews. Although extensive efforts were made to reduce its impact, this familiarity may have influenced these participants’ responses. Finally, although participants with lived experience were involved in study planning, they had no role in data analysis. We attempted to address this by member-checking results with study participants. However, involvement of a lived experience researcher during data analysis may have contributed important additional insights not reflected within the analysis presented here.

## Conclusion

Conceptualisation of patient-centred AIWG management by research participants contrasted significantly with current management algorithms. Participants reported that existing guidance is oversimplified, lacks the specificity and scope required, and endorses a ‘one-size-fits-all’ management approach to an extensively heterogenous side-effect. Management algorithms that prioritise dietary and lifestyle interventions and endorse restricted access to pharmacological adjuncts reflect neither empirical evidence nor patient preference. They can inadvertently contribute to internalised weight bias among patients by framing management as primarily an individual's responsibility. Participants expressed a preference for collaborative AIWG management and guidance that prioritises early intervention using the range of evidence-based management interventions, tailored according to AIWG risk, patient ability and preferences. Use of this research in guideline development will ensure that recommendations are relevant and applicable, and will provide opportunities to maximise the impact of recommendations in practice for those funding, providing and using services. In practice, participants reported that clinicians overestimate AIWG manageability using dietary and lifestyle changes, and reported barriers to accessing endorsed alternative management interventions. This included either a change in antipsychotic and/or prescriptions for pharmacological adjuncts. Thus, aside from improved guidance informed by lived experience, extensive efforts will be required to improve uptake of patient-centred and evidence-based AIWG management recommendations within psychiatric services. This should include efforts to address both explicit and implicit sources of weight bias among healthcare providers.

## Supporting information

Fitzgerald et al. supplementary material Fitzgerald et al. supplementary material

## Data Availability

The data that support the findings of this study are not publicly available or available on request, as doing so would violate the agreement with which participants consented to partake in this study and would compromise the privacy of the research participants. Anonymised excerpts have been provided as part of this manuscript with explicit participant consent.

## References

[1] McCrone P, Mosweu I, Yi D, Ruffell T, Dalton B, Wykes T. Patient preferences for antipsychotic drug side effects: a discrete choice experiment. Schizophr Bull Open 2021; 2(1): sgab046.

[2] Velligan DI, Sajatovic M, Hatch A, Kramata P, Docherty JP. Why do psychiatric patients stop antipsychotic medication? A systematic review of reasons for nonadherence to medication in patients with serious mental illness. Patient Prefer Adherence 2017; 3(11): 449–68. 10.2147/PPA.S124658 PMC5344423 28424542

[3] Bak M, Fransen A, Janssen J, van Os J, Drukker M. Almost all antipsychotics result in weight gain: a meta-analysis. PLoS One 2014; 9(4): e94112. 10.1371/journal.pone.0094112 PMC3998960 24763306

[4] Firth J, Siddiqi N, Koyanagi A, Siskind D, Rosenbaum S, Galletly C, et al. The Lancet Psychiatry Commission: a blueprint for protecting physical health in people with mental illness. Lancet Psychiatry 2019; 6(8): 675–712. 31324560 10.1016/S2215-0366(19)30132-4

[5] Dandona R. Mind and body go together: the need for integrated care. Lancet Psychiatry 2019; 6(8): 638–9. 31324559 10.1016/S2215-0366(19)30251-2

[6] Gangopadhyay A, Ibrahim R, Theberge K, May M, Houseknecht KL. Non-alcoholic fatty liver disease (NAFLD) and mental illness: mechanisms linking mood, metabolism and medicines. Front Neurosci 2022; 16: 1042442. 36458039 10.3389/fnins.2022.1042442 PMC9707801

[7] Lee K, Akinola A, Abraham S. Antipsychotic-induced weight gain: exploring the role of psychiatrists in managing patients’ physical health challenges, current options and direction for future care. BJPsych Bull 2024; 48(1): 24–9. 37165776 10.1192/bjb.2023.29 PMC10801410

[8] Holt RIG. The management of obesity in people with severe mental illness: an unresolved conundrum. Psychother Psychosom 2019; 88(6): 327–32. 31587002 10.1159/000503835

[9] Fitzgerald I, Crowley EK, Byrne A, O'Connell J, Ensor J, Ni Dhubhlaing C, et al. Predicting antipsychotic-induced weight gain in first episode psychosis – a protocol for a field-wide systematic review of prognostic factor studies. Int J Clin Trials 2022; 9(4): 300–13.

[10] de Boer N, Cahn W. Antipsychotic-induced weight gain: is the weight over? New guidelines needed. Acta Psychiatr Scand 2022; 146(3): 185–9. 35951775 10.1111/acps.13485

[11] World Health Organization (WHO). Management of Physical Health Conditions in Adults with Severe Mental Disorders: WHO Guidelines. WHO, 2018 (https://www.ncbi.nlm.nih.gov/books/NBK534487/). 30507109

[12] Cooper SJ, Reynolds GP, Barnes T, England E, Haddad PM, Heald A, et al. BAP guidelines on the management of weight gain, metabolic disturbances and cardiovascular risk associated with psychosis and antipsychotic drug treatment. J Psychopharmacol 2016; 30(8): 717–48. 27147592 10.1177/0269881116645254

[13] Taylor D, Gaughran F, Pillinger T. The Maudsley Practice Guidelines for Physical Health Conditions in Psychiatry. Wiley, 2021.

[14] Siemieniuk R, Guyatt G. What is GRADE? BMJ Best Practice, 2021 (https://bestpractice.bmj.com/info/toolkit/learn-ebm/what-is-grade/).

[15] Downe S, Finlayson KW, Lawrie TA, Lewin SA, Glenton C, Rosenbaum S, et al. Qualitative evidence synthesis (QES) for guidelines: paper 1 – using qualitative evidence synthesis to inform guideline scope and develop qualitative findings statements. Health Res Policy Syst 2019; 17(1): 76. 31391057 10.1186/s12961-019-0467-5 PMC6686511

[16] Vancampfort D, Firth J, Correll CU, Solmi M, Siskind D, De Hert M, et al. The impact of pharmacological and non-pharmacological interventions to improve physical health outcomes in people with schizophrenia: a meta-review of meta-analyses of randomized controlled trials. World Psychiatry 2019; 18(1): 53–66. 30600626 10.1002/wps.20614 PMC6313230

[17] Agarwal SM, Stogios N, Ahsan ZA, Lockwood JT, Duncan MJ, Takeuchi H, et al. Pharmacological interventions for prevention of weight gain in people with schizophrenia. Cochrane Database Syst Rev 2022; 10(10): CD013337. 36190739 10.1002/14651858.CD013337.pub2 PMC9528976

[18] National Institute of Clinical Excellence (NICE). Surveillance Proposals and Evidence – Psychosis and Schizophrenia in Children and Young People: Recognition and Management Guidance. NICE, 2023 (https://www.nice.org.uk/guidance/cg155/resources/2023-exceptional-surveillance-of-psychosis-and-schizophrenia-in-children-young-people-and-adults-nice-guidelines-cg155-and-cg178-13244105197/chapter/Surveillance-proposals?tab=evidence).

[19] Speyer H, Westergaard C, Albert N, Karlsen M, Stürup AE, Nordentoft M, et al. Reversibility of antipsychotic-induced weight gain: a systematic review and meta-analysis. Front Endocrinol 2021; 12: 577919. 10.3389/fendo.2021.577919 PMC8355990 34393989

[20] Khaity A, Mostafa Al-Dardery N, Albakri K, Abdelwahab OA, Hefnawy MT, Yousef YAS, et al. Glucagon-like peptide-1 receptor-agonists treatment for cardio-metabolic parameters in schizophrenia patients: a systematic review and meta-analysis. Front Psychiatry 2023; 14: 1153648. 37215670 10.3389/fpsyt.2023.1153648 PMC10196269

[21] Castle DJ, Galletly CA, Dark F, Humberstone V, Morgan VA, Killackey E, et al. The 2016 Royal Australian and New Zealand College of Psychiatrists guidelines for the management of schizophrenia and related disorders. Aust N Z J Psychiatry 2016; 50(5): 410–72. 27106681 10.1177/0004867416641195

[22] Fitzgerald I, O'Connell J, Keating D, Hynes C, McWilliams S, Crowley EK. Metformin in the management of antipsychotic-induced weight gain in adults with psychosis: development of the first evidence-based guideline using GRADE methodology. Evid Based Ment Health 2021; 25(1): 15–22. 34588212 10.1136/ebmental-2021-300291 PMC8788031

[23] Puhl RM, Heuer CA. The stigma of obesity: a review and update. Obesity 2009; 17(5): 941–64. 19165161 10.1038/oby.2008.636

[24] Brown A, Flint SW, Batterham RL. Pervasiveness, impact and implications of weight stigma. EClinicalMedicine 2022; 47: 101408. 35497065 10.1016/j.eclinm.2022.101408 PMC9046114

[25] Bailey E. More than a Number – Centre for Mental Health, Rethink Mental Illness and the Association of Mental Health Providers. Equally Well, 2020 (https://equallywell.co.uk/resources/more-than-a-number/).

[26] Mizock L. The double stigma of obesity and serious mental illnesses: promoting health and recovery. Psychiatr Rehabil J 2012; 35(6): 466–9. 23276241 10.1037/h0094581

[27] Ryan L, Coyne R, Heary C, Birney S, Crotty M, Dunne R, et al. Weight stigma experienced by patients with obesity in healthcare settings: a qualitative evidence synthesis. Obes Rev [Epub ahead of print] 2 Aug 2023. Available from: 10.1111/obr.13606. 37533183

[28] Tong A, Sainsbury P, Craig J. Consolidated criteria for reporting qualitative research (COREQ): a 32-item checklist for interviews and focus groups. Int J Qual Health Care 2007; 19(6): 349–57. 17872937 10.1093/intqhc/mzm042

[29] Bradshaw C, Atkinson S, Doody O. Employing a qualitative description approach in health care research. Glob Qual Nurs Res 2017; 15(4): 2333393617742282. 10.1177/2333393617742282 PMC5703087 29204457

[30] Doyle L, McCabe C, Keogh B, Brady A, McCann M. An overview of the qualitative descriptive design within nursing research. J Res Nurs 2020; 25(5): 443–55. 34394658 10.1177/1744987119880234 PMC7932381

[31] Braun V, Clarke V. To saturate or not to saturate? Questioning data saturation as a useful concept for thematic analysis and sample-size rationales. Qual Res Sport Exerc Health 2021; 13(2): 201–16.

[32] Roller MR, Lavrakas PJ. Applied Qualitative Research Design: A Total Quality Framework Approach. The Guildford Press, 2015.

[33] Byrne D. A worked example of Braun and Clarke's approach to reflexive thematic analysis. Qual Quant 2022; 56(3): 1391–412.

[34] Braun V, Clarke V. Reflecting on reflexive thematic analysis. Qual Res Sport Exerc Health 2019; 11(4): 589–97.

[35] Rocha-González HI, De la Cruz-Álvarez LE, Kammar-García A, Canizales-Quinteros S, Huerta-Cruz JC, Barranco-Garduño LM, et al. Weight loss at first month and development of tolerance as possible predictors of 30 mg phentermine efficacy at 6 months. J Pers Med 2021; 11(12): 1354. 34945825 10.3390/jpm11121354 PMC8707701

[36] Waite F, Langman A, Mulhall S, Glogowska M, Hartmann-Boyce J, Aveyard P, et al. The psychological journey of weight gain in psychosis. Psychol Psychother 2022; 95(2): 525–40. 35137519 10.1111/papt.12386 PMC9304181

[37] Speyer H, Jakobsen AS, Westergaard C, Nørgaard HCB, Jørgensen KB, Pisinger C, et al. Lifestyle interventions for weight management in people with serious mental illness: a systematic review with meta-analysis, trial sequential analysis, and meta-regression analysis exploring the mediators and moderators of treatment effects. Psychother Psychosom 2019; 88(6): 350–62. 31522170 10.1159/000502293

[38] De Silva VA, Suraweera C, Ratnatunga SS, Dayabandara M, Wanniarachchi N, Hanwella R. Metformin in prevention and treatment of antipsychotic induced weight gain: a systematic review and meta-analysis. BMC Psychiatry 2016; 16(1): 341. 27716110 10.1186/s12888-016-1049-5 PMC5048618

[39] Brandl EJ, Frydrychowicz C, Tiwari AK, Lett TAP, Kitzrow W, Büttner S, et al. Association study of polymorphisms in leptin and leptin receptor genes with antipsychotic-induced body weight gain. Prog Neuropsychopharmacol Biol Psychiatry 2012; 38(2): 134–41. 22426215 10.1016/j.pnpbp.2012.03.001

[40] Fitzgerald I, Sahm LJ, Byrne A, O'Connell J, Ensor J, Ní Dhubhlaing C, et al. Predicting antipsychotic-induced weight gain in first episode psychosis – a field-wide systematic review and meta-analysis of non-genetic prognostic factors. Eur Psychiatry 2023; 66(1): e42. 37278237 10.1192/j.eurpsy.2023.2417 PMC10305761

[41] Lamontagne-Godwin F, Burgess C, Clement S, Gasston-Hales M, Greene C, Manyande A, et al. Interventions to increase access to or uptake of physical health screening in people with severe mental illness: a realist review. BMJ Open 2018; 8(2): e019412. 10.1136/bmjopen-2017-019412 PMC5829934 29440160

[42] Breen C, O'Connell J, Geoghegan J, O'Shea D, Birney S, Tully L, et al. Obesity in adults: a 2022 adapted clinical practice guideline for Ireland. Obes Facts 2022; 15(6): 736–52. 36279848 10.1159/000527131 PMC9801383

[43] Robles B, Kuo T, Galván A. Understanding the neuroscience underpinnings of obesity and depression: implications for policy development and public health practice. Front Public Health 2021; 9: 714236. 34490195 10.3389/fpubh.2021.714236 PMC8417597

[44] Zhang JP, Lencz T, Zhang RX, Nitta M, Maayan L, John M, et al. Pharmacogenetic associations of antipsychotic drug-related weight gain: a systematic review and meta-analysis. Schizophr Bull 2016; 42(6): 1418–37. 27217270 10.1093/schbul/sbw058 PMC5049532

