# Supplementary material for: Informing the development of antipsychotic-induced weight gain management guidance: patient experiences and preferences – qualitative descriptive study
Source: BJPsych Open. 2024 Aug 1;10(5):e136. doi: 10.1192/bjo.2024.725 (PMC11698206; doi:10.1192/bjo.2024.725)
Supplement: Fitzgerald et al. supplementary material [file S2056472424007257sup001.pdf]

Informing the development of antipsychotic-induced weight gain management guidance: patient experiences, preferences and expectations - a qualitative descriptive study

Table of Contents

Part 1 – Criteria for reporting qualitative health research checklist (COREQ) checklist 2

Part 2 – Interview guide (Version 7)..5

Part 3 – Interviewer characteristics (COREQ Item No. 8)...10

Part 1 – Criteria for reporting qualitative health research checklist (COREQ) checklist

Consolidated criteria for reporting qualitative studies (COREQ): 32-item checklist

**No. Item**	Guide questions/description	Reported on Page #
**Domain 1: Research team and reflexivity**		
Personal Characteristics		
1. Interviewer/facilitator	Which author/s conducted the interview or focus group?	3
2. Credentials	What were the researcher's credentials? E.g. PhD, MD	19
3. Occupation	What was their occupation at the time of the study?	3
4. Gender	Was the researcher male or female?	3
5. Experience and training	What experience or training did the researcher have?	3
Relationship with participants		
6. Relationship established	Was a relationship established prior to study commencement?	3
7. Participant knowledge of the interviewer	What did the participants know about the researcher? e.g. personal goals, reasons for doing the research	3
8. Interviewer characteristics	What characteristics were reported about the inter viewer/facilitator? e.g. Bias, assumptions, reasons and interests in the research topic	Supplementary appendix part 3

**Domain 2: study design**		
Theoretical framework		
9. Methodological orientation and Theory	What methodological orientation was stated to underpin the study? e.g. grounded theory, discourse analysis, ethnography, phenomenology, content analysis	3
Participant selection		
10. Sampling	How were participants selected? e.g. purposive, convenience, consecutive, snowball	4
11. Method of approach	How were participants approached? e.g., face-to-face, telephone, mail, email	3
12. Sample size	How many participants were in the study?	5
13. Non-participation	How many people refused to participate or dropped out? Reasons?	n/a as participants self-referred following advertisement
Setting		
14. Setting of data collection	Where was the data collected? e.g. home, clinic, workplace	3
15. Presence of non-participants	Was anyone else present besides the participants and researchers?	No
16. Description of sample	What are the important characteristics of the sample? e.g. demographic data, date	5
Data collection		
17. Interview guide	Were questions, prompts, guides provided by the authors? Was it pilot tested?	4
18. Repeat interviews	Were repeat interviews carried out? If yes, how many?	No
19. Audio/visual recording	Did the research use audio or visual recording to collect the data?	3
20. Field notes	Were field notes made during and/or after the interview or focus group?	3
21. Duration	What was the duration of the inter views or focus group?	3
22. Data saturation	Was data saturation discussed?	4

23. Transcripts returned	Were transcripts returned to participants for comment and/or correction?	4
**Domain 3: analysis and findings**		
Data analysis		
24. Number of data coders	How many data coders coded the data?	4
25. Description of the coding tree	Did authors provide a description of the coding tree?	4
26. Derivation of themes	Were themes identified in advance or derived from the data?	4
27. Software	What software, if applicable, was used to manage the data?	4
28. Participant checking	Did participants provide feedback on the findings?	5
Reporting		
29. Quotations presented	Were participant quotations presented to illustrate the themes/findings? Was each quotation identified? e.g. participant number	6-15
30. Data and findings consistent	Was there consistency between the data presented and the findings?	6-18
31. Clarity of major themes	Were major themes clearly presented in the findings?	5+11
32. Clarity of minor themes	Is there a description of diverse cases or discussion of minor themes?	Only major themes found

Part 2 – Interview guide (Version 7)

Topic	Question(s)	Prompt(s)
1. Gaining weight from antipsychotic treatment	Q1. Could you tell me about your experience with weight gain caused by your antipsychotic medication?<br><br>Q2. Can you give me some words to describe your experience with gaining weight from antipsychotics?	- When did you first notice weight gain was a side effect of your antipsychotic?<br><br>- How much weight did you gain?<br><br>- Do you continue to gain weight?<br><br>- How did you learn weight gain was a side effect of your medication?<br><br>- How has this weight gain impacted you?<br><br>- When this happens, is this something that occupies a lot of your time thinking about, and if so, why do you think that is? (new)
2. Managing antipsychotic-induced weight gain personally	Q1. Could you tell me about how you manage this extra weight yourself? This can include any option or method that you have tried.<br><br>Q2. Are there any methods that you found particularly helpful (or unhelpful) to reduce or prevent further weight gain?<br><br>Q3. If any methods were particularly helpful/unhelpful - why do you think that method(s) was successful/unsuccessful?<br><br>Q3. How does how you manage weight gain change depending on your mental health status?	- What methods (if any) did you use to try to lose, or reduce further weight gain, caused by taking your antipsychotic?<br><br>- Have you tried diet and lifestyle options to lose weight? If so, could you tell me about what you did?<br><br>- Have you tried to change your medications to try and reverse?<br><br>- Have you ever tried any medications that could be bought without a prescription or online to

	Q4. For those who have tried medications - How did the medication help you to manage your weight? Was this different to other approaches you had tried e.g., dietary changes? If so, how?	manage weight gain caused by antipsychotics?
3. Experience of managing antipsychotic-induced weight gain – acceptability of interventions provided by healthcare services	Q1. What option(s) have been suggested to you by healthcare providers/services as ways to reduce weight gain caused by antipsychotics?<br><br>Q2. At what stage of treatment were support options offered to you?<br><br>Q3. From question 1 - how suitable or acceptable did you feel those choices were?<br><br>Q4. If support options were offered to you to reduce weight gain from antipsychotics, what follow up was provided to you? This might include asking how well an intervention worked or whether other options should be tried.<br>Q5. Do you think that your physical healthcare was prioritized in your experience of mental health services?	- Were a range of management options provided to you?<br><br>- What lifestyle advice was offered to you?<br><br>- What dietary advice was offered to you?<br><br>- Have you been offered additional medications to manage your weight?<br><br>- If one management option was not suitable, were you offered an alternative management option?<br><br>- How do you feel about the timing at which interventions were offered?<br><br>- Have you sought/been offered support from a specialist weight management service?<br><br>- In the case of dietary advice, were you offered personal/group/general advice?
4. Experience of managing antipsychotic-induced weight gain within the healthcare system –	Q1. Which healthcare providers did you ask for support with managing weight gain caused by antipsychotics? This could include your general practitioner	- Did you ask for support (management options) from healthcare providers with managing weight gain

experience with healthcare providers	(GP) or someone from your mental healthcare team.<br><br>Q2. Can you give me some words to describe this experience of speaking with healthcare providers about managing weight gain caused by antipsychotics?<br><br>Q3. Who do you feel is responsible for managing weight gain caused by antipsychotics?<br><br>Q4. Do you feel like you were adequately supported (across any setting) in managing this side effect?	caused by antipsychotics or was it offered to you first?<br><br>- Did you ask the person(s) who prescribes your antipsychotic medication for support with managing your weight?<br><br>- How did the management experiences with healthcare professionals around this side effect leave you feeling? (new)<br><br>- Did you ask or discuss weight gain caused by antipsychotics with someone outside your mental health team, including a GP, private dietician etc.<br><br>- If you didn't ask for help or support, why was this?<br><br>- Is discussing your weight with those who look after your mental health or general healthcare provider something you feel comfortable doing?<br><br>- How did it feel to ask for help with managing weight gain caused by antipsychotics?<br><br>- Did you feel that you were listened to when you asked for support with managing your weight?<br><br>- Was there any difference n care provision approach by those who did and did not have experience with AIWG e.g., GP vs. psychiatrist (new)

5. Current standard of management - hierarchical management of antipsychotic-induced weight gain	Currently what is recommended to those who work in healthcare when helping service users manage weight gain caused by antipsychotics is to recommend what is called a 'tiered approach'. This means that first we should offer diet and lifestyle advice to help you manage your weight, then consider changing antipsychotic medication and finally, adding in an extra medication to help with weight loss.<br><br>Q1. Was this type of approach offered to you when managing weight gain caused by antipsychotics?<br><br>Q2. How appropriate do you think this approach is?<br><br>Q3. How do you feel about taking an extra medication to treat or prevent current weight gain?<br><br>Q4. When should medications to manage weight gain caused by antipsychotics be considered? (re-phrased)<br><br>Q5. How do you feel about a combination of approaches being used to manage weight gain caused by antipsychotics<br><br>Q6. Some new medications that are available to reduce weight gain caused by antipsychotics are medications that need to be injected once a day or once a week. How do you feel about this option?	- Do you think diet and lifestyle approaches should be tried by everyone before other options, like switching antipsychotics, or trying a medication to reduce appetite, are tried?<br><br>- Did you discuss switching antipsychotic medication with a healthcare provider to manage weight gain caused by antipsychotics?<br><br>- Were you ever offered the use of an additional medication to help manage your weight?<br><br>- Should we apply the same management approach in everyone?<br><br>- Would you prefer to wait to take an additional tablet to manage your weight until weight gain had occurred?<br><br>- Would you prefer to wait to take an additional tablet to manage your weight until other options have failed, for example trying a new diet or increasing the amount of exercise you do?

6. Service user attitude, values, and preferences on AIWG management	Q1. What does improved management of this side effect look like to you?<br><br>Q2. What would be the most important thing(s) a service should provide to help service users manage weight gain caused by antipsychotics?<br><br>Q3. How could healthcare providers improve their communication with service users around weight gain caused by antipsychotics?<br><br>Q4. When considering whether to take an antipsychotic that is associated with weight gain, what information about this side effect would you want to know?<br><br>Q5. Do you think you can be an active partner in deciding what medication to take if you are experiencing mental health symptoms? What does this depend on?	- Picture if you were to start again with taking an antipsychotic and were experiencing unwanted weight gain, what does improved management look like to you?<br><br>- How often should weight be checked?<br><br>- How do you feel about being weighed or weighing yourself?<br><br>- Should the intensity of supports you are offered change depending on your mental health symptoms?<br><br>- When should we speak to service users about weight gain that antipsychotics can cause?<br><br>- How do you feel about interacting with a specialist in exercise like you would interacting with a specialist in food i.e., dietician?<br><br>- When you are unwell, do you think you can engage in conversations at the point of prescribing around side effects like weight gain?

Closing – I'd like to finish the interview by asking you if there is anything else that you would like to mention that we haven't spoken about thus far?

Part 3 – Interviewer characteristics (COREQ Item No. 8): What characteristics were reported about the interviewer/facilitator? e.g. Bias, assumptions, reasons and interests in the research topic

Each interview participant was given an overview of the researcher conducting the interviewer (IF) interest in the topic of antipsychotic-induced weight gain (AIWG) management. This includes information about their clinical role in psychiatry and work on a PhD project focussed on improving the management of antipsychotic-induced weight gain. IF discussed their interest in this work and why they conceptualised the PhD project with the research team and this qualitative work specifically. IF was aware of issues highlighted by patients they had previously worked with in clinical practice about AIWG and suboptimal management practices, and therefore wanted to work with those with lived experience of AIWG to improve management. This was explained to participants who had self-referred to the study following advertisements. Five participants who were aware of the research study due to previous interactions with IF were aware of their interest in improving the management of AIWG as clinical interactions were typically based in this area. This group of participants were asked prior to interview commencement to adopt the position that the researcher conducting interviews was not aware of their history as other team members would review their anonymised transcripts. All participants were encouraged to share their views openly.
